# Beyond REM: A New Approach to the Use of Image Classifiers for the Management of 6G Networks

**DOI:** 10.3390/s23177494

**Published:** 2023-08-29

**Authors:** Eduardo Baena, Sergio Fortes, Francisco Muro, Carlos Baena, Raquel Barco

**Affiliations:** Instituto de Telecomunicación (TELMA), CEI Andalucía TECH, E.T.S. Ingeniería de Telecomunicación, Universidad de Málaga, 29010 Málaga, Spain

**Keywords:** 5G, 6G, image classification, REM, location-aware

## Abstract

The management of cellular networks, particularly within the environment rapidly advancing to 6G, presents considerable challenges due to the highly dynamic radio environment. Traditional tools such as Radio Environment Maps (REMs) have proven inadequate for real-time network changes, underlining the need for more sophisticated solutions. In response to these challenges, this work introduces a novel approach that harnesses the unprecedented power of state-of-the-art image classifiers for network management. This method involves the generation of Network Synthetic Images (NSIs), which are enriched heat maps that precisely reflect varying cellular network operating states. Created from user location traces linked with Key Performance Indicators (KPIs), NSIs are strategically designed to meet the intricate demands of 6G networks. This research delves deep into a comprehensive analysis of the diverse factors that could potentially impact the successful application of this methodology in the realm of 6G. The results from this investigation, coupled with a comparative assessment against traditional REM usage, emphasize the superior performance of this innovative method. Additionally, a case study involving an automatic network diagnosis scenario validates the effectiveness of this approach. The findings reveal that a generic Convolutional Neural Network (CNN), one of the most powerful tools in the arsenal of modern image classifiers, delivers enhanced performance, even with a reduced demand for positioning accuracy. This contributes significantly to the real-time, robust management of cellular networks as we transition into the era of 6G.

## 1. Introduction

The rapid progression of mobile communication technologies has induced several advancements that fundamentally transform methods of communication, work, and lifestyle. The transition from 5G to 6G networks is anticipated to bring transformative changes, introducing ultra-reliable low-latency communication, enhanced mobile broadband, and massive machine-type communication capabilities [1]. Within this evolving landscape, the concept of zero-touch networks has emerged as a critical paradigm designed to enhance the efficiency, flexibility, and scalability of complex systems while minimizing operational costs and human intervention [2,3].

The zero-touch (ZT) network paradigm symbolizes the automation of network operations, management, and optimization processes. Powered by intelligent and autonomous systems, these are crucial for managing the scale and complexity of 5G and b5G networks, thereby ensuring optimal performance, resource utilization, and user experience. A critical element in the realization of zero-touch networks is real-time decision support tools that enable data-driven decision-making and swift adaptation to the dynamic and heterogeneous radio environment.

Traditionally, tools such as coverage maps and Radio Frequency (RF) maps have been relied upon for network deployment, playing key roles in site selection, capacity planning, and interference management. These tools often fail to represent the dynamic and diverse radio environment adequately. To address this, Radio Environment Maps (REMs) were introduced [4]. Yet, despite their evolution, REMs face limitations in supporting zero-touch network operations due to their limited capacity to accurately reflect the real-time complexity of the wireless environment [5].

In parallel with the advancement of 5G standards, data processing systems and Machine Learning (ML) algorithms have significantly evolved [6,7]. The integration of mature positioning technologies has opened up a new path. In this context, localization of User Equipment (UEs) is critical for identifying, locating, and classifying network failures. Ubiquitous UE localization, both outdoors and indoors, is anticipated, facilitated by information from global navigation satellite systems (GNSS) and the use of cellular-based precise location systems [8]. This localization data is critical to the context-aware paradigm, where variables impacting cellular performance, such as user distributions, are considered for network management.

The integration of localized information into cellular management, particularly fault diagnosis, presents a series of challenges [9]. The collection and formatting of heterogeneous data generated by various network elements can be complex, and the analysis of such data in live systems introduces further difficulties. Understanding the interconnectivity of faults originating from multiple points in the network and deciphering causality in such extensive, complex systems can be daunting.

Meanwhile, advancements in Deep Learning (DL) mechanisms, particularly Convolutional Neural Networks (CNNs), have been driven by their applications in fields such as automated driving, security, and health [10]. These fields heavily depend on image and video recognition and classification tasks. While DL algorithms have begun to penetrate autonomic network literature, there is a need for more comprehensive approaches that utilize image classifiers for this purpose [11].

Image classifiers, proficient in capturing, processing, and analyzing visual data, hold the potential to model the complexities and temporal variations of wireless networks effectively. They can reveal hidden cross-relations among variables, thereby reducing the need for ultra-precise location methods and opening up new opportunities for advanced image processing algorithms in mobile network management.

Although the methodologies for creating REMs are rapidly evolving, they face significant challenges. These include the dynamic nature of the wireless environment, inherent uncertainty, temporal dynamics, and the robustness and efficiency of various algorithms. Such challenges indicate room for considerable improvements and innovative approaches in the future.

In response to these limitations, this work presents a proof-of-concept (PoC) that sets the foundation for the application of advanced image processing algorithms in mobile network management. A novel framework that utilizes DL image classification algorithms in zero-touch network tasks is introduced. This framework presents the concept of Network Synthetic Images (NSIs), an innovative approach to synthesizing information about network operations in a specific area. In the NSI application and generation processes, aspects of image classifiers such as convolutional neural networks, transfer learning, and data augmentation techniques are carefully considered. These techniques, which have proven effective across a wide range of applications, are expected to impact this context [12] significantly.

The key contributions of this work are:

- The proposition of a novel Network Synthetic Images (NSI) framework transforms the process of synthesizing and interpreting information in network operations. This innovative framework leverages the capabilities of image classifiers to detect hidden patterns and uncover unforeseen effects often beyond the reach of human perception and traditional REMs.
- A comprehensive discussion of the essential aspects required to effectively apply image classifiers to NSIs for network management tasks, including an in-depth exploration of CNNs.
- An evaluation of the proposed NSI framework using a generic CNN and comparing it against state-of-the-art REM image classifier methods. The results highlight the superior performance of the proposed framework, with improved F1 and accuracy scores achieved, even with coarser time and spatial resolution, effectively reducing the training time.
- An outline of practical implementation aspects and challenges of integrating this methodology into future 6G network technologies. This exploration provides insight into potential obstacles, laying the groundwork for future research to address these challenges.

In this way, the present work is organized as follows. Section 2 presents previous works related to REMs and their usage in cellular network automation. Subsequently, the proposed Network state Synthetic image creation framework for using image classification algorithms is presented in Section 3, describing the details of the proposed NSIs approach and the rest of the system’s functionalities. The proposed system is evaluated in its application to supervised Fault diagnosis in Section 4. Finally, Section 5 summarizes the findings and identifies future research lines.

## 2. Related Work

The transition in mobile network environments, specifically the shift towards 6G networks, necessitates adaptive decision support tools [1]. Radio Environment Maps (REMs) play a crucial role in cellular network management. However, the capacity of REMs to accommodate rapidly changing conditions has become increasingly strained [13].

Traditionally, REMs were generated through the deployment of numerous sensors across a specific area, which collected Received Signal Strength (RSS) measurements to create a coverage map. Unfortunately, this static approach was found to be insufficient for effectively capturing the dynamic nature of radio environments.

Despite these limitations, REMs have proven to be key tools in efficient spectrum management [13,14,15], a necessity in light of the impending spectrum scarcity accompanying the rise of 6G networks. Achievements in REM accuracy have been realized by using synthetic images [16] and spatial statistics in combination with Bayesian hierarchical models [17,18]. However, the inherent unpredictability and complexity of wireless environments continue to pose significant challenges.

A broad spectrum of publications has focused on REM construction and implementation. Various machine learning (ML) mechanisms have been employed for purposes such as updating and construction, among others. These strategies include traditional clustering techniques such as K-means and DBSCAN [13,14], Gaussian mixture models [15], and Bayesian networks [19]. Advanced strategies for REM updating, such as Siamese neural networks and attention mechanisms, have also been incorporated [20]. Table 1 offers a detailed survey of various ML-based strategies for REMs in current literature, summarizing the applications, data sources, and identified gaps in each method.

The scope of REM applications has recently been expanded, with a focus on optimizing network operations. Examples include the use of REMs in secrecy-energy efficient hybrid beamforming for satellite-terrestrial integrated networks [12], rate-splitting multiple access schemes for IoT support in satellite and aerial-integrated networks [24], and joint beamforming design for refracting Reconfigurable Intelligent Surfaces (RIS)-aided hybrid networks [25]. These studies highlight the versatile application of REMs across various network operations.

In anticipation of 6G networks, this research introduces an innovative technique that employs geolocated user traces to advance traditional REMs into Network State Synthetic Images (NSIs), converting various cellular network operating states into comprehensive heat maps. While synthetic image generation for REMs has been previously considered [16], the application of deep learning (DL) techniques to process geolocated traces into an image-like format for 6G networks remains unexplored. Past attempts to utilize image classifiers for network fault detection were hindered by reliance on inadequately processed images. This research, conversely, advocates the creation of NSIs using image classifiers for network management, representing a significant advancement in the field.

## 3. Proposed System

**Figure 1:**
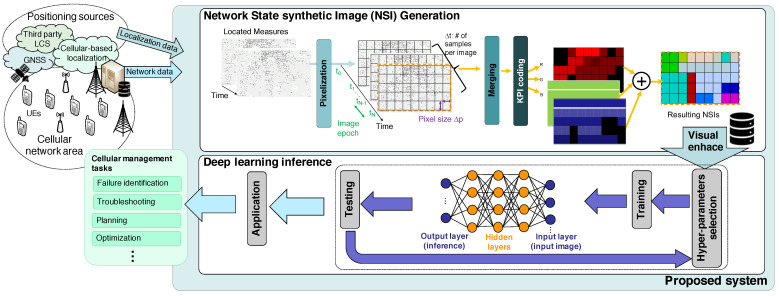
Framework for NSI use and application.

This section presents a broad framework aimed at addressing the current limitations in applying DL image classifier methods for cellular management, as outlined in Figure 1. Initially, the system collects UE traces that include both network and localization data. Network data—such as radio conditions and the performance experienced by the UEs—are directly available as part of the network’s management and control plane structure. These measurements, in combination with the location data, are hereinafter referred to as Localized Measurements (LMs). In contrast, localization data, increasingly integrated into cellular network management and control planes, can be sourced from cellular network-based localization (directly available by the operator), Global Navigation Satellite Systems (GNSS), or additional third-party Localization Services (LCS) [26].

The ensuing subsections provide an in-depth description of the proposed method for both the generation of NSIs and their subsequent DL inference analysis, especially highlighting its application to failure diagnosis.

After data acquisition, the next step involves processing this information to create the proposed Network Synthetic Images (NSI). These image-like data structures are integral to applying DL image analysis mechanisms. Subsequently, these algorithms, based on the patterns identified in the NSIs, enable the execution of various network management tasks such as failure identification/diagnosis, early error state detection, and network planning and optimization.

### 3.1. Generation of Synthetic Network Images

A transformation is proposed from a dataset of systematically collected geotagged and timestamped traces into synthetic images. These images encapsulate the network state in a structured manner for a geographical area, as illustrated in Figure 1. Spatial and temporal resolutions are key aspects of this representation, defining the level of detail captured both in space and time.

The first step of this process is to define a specific spatial and temporal resolution. This defines the size of the pixels in the synthetic image and the time period each image will cover. The geographical location of each UE at a given interval, denoted as $Loc_{UE_{i}}\left(T\right)$, is expressed as a two-dimensional spatial vector containing x and y coordinates, respectively. Along with this location information, a set of measurements of different Key Performance Indicators is collected:

(1) $$LM_{UE_{i}}\left(T\right)=\left\{Loc_{UE_{i}}\left(T\right),KPI_{UE_{i}}^{1}\left(T\right),KPI_{UE_{i}}^{2}\left(T\right),\ldots ,KPI_{UE_{i}}^{k}\left(T\right)\right\}$$

The pixelization step defines the spatial resolution of the scenario by splitting the geographical area of interest *A* into a grid of pixels $P_{i,j}$. The size of each pixel, denoted Δp, should be based on the specific application needs:

(2) $$A=\left\{P_{i,j}|i=1,\ldots ,n;\;j=1,\ldots ,m\right\}$$

The set of LMs within each pixel, which contains multiple measurements from the UEs in its vicinity during the period of interest, is defined as:

(3) $$LM_{P_{i,j}}=\left\{LM_{UE_{k}}\left(T\right)|Loc_{UE_{k}}\left(T\right)\in P_{i,j},UE_{k}\in U_{T},T\in T_{t}\right\}$$

In the MergeLM (merging) step, statistical figures or a linear combination are used to summarize the measurements within each pixel. For instance, the average measurement value ${\bar{KPI}}_{P_{i,j}}$ for each pixel is calculated as:

(4) $${\bar{KPI}}_{P_{i,j}}=\frac{1}{|LM_{P_{i,j}}|}\sum_{LM_{UE_{k}}\left(T\right)\in LM_{P_{i,j}}}KPI_{UE_{k}}\left(T\right)$$

**Figure 2:**
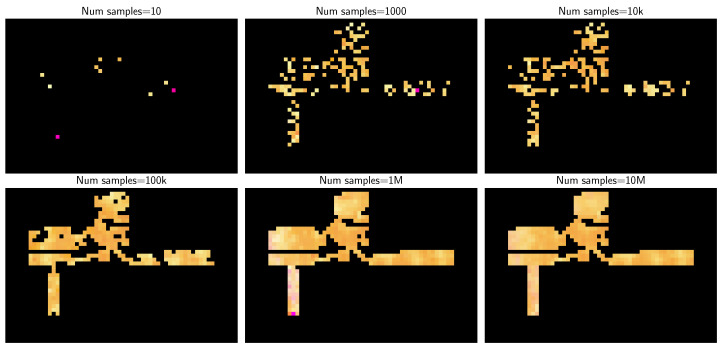
NSI with RSRP, RSRQ, and SINR mapped as RGB with varying number of samples (e.g., time resolution).

Balancing between high resolution, which allows for a detailed state view of the network but requires more data and computational resources, and low resolution, which offers less detail but is more resource-efficient, is crucial. Factors such as the number of monitored users, their distribution, and movements within the area significantly influence this balance. Figure 2 illustrates how the number of samples (time resolution) influences the construction of an NSI (Figure 1) for an operational consistent state.

The process of LM coding in this context could be formulated as the transformation of each pixel $P_{i,j}$, with its set of local measurements $LM_{P_{i,j}}$ and their aggregated metrics such as ${\bar{KPI^{1}}}_{P_{i,j}}$, ${\bar{KPI^{2}}}_{P_{i,j}}$, and ${\bar{KPI^{3}}}_{P_{i,j}}$, into a color representation (i.e., in the Red Green Blue (RGB) format).

In this case, each color component in RGB corresponds to a different Key Performance Indicator (KPI). For instance, ${\bar{KPI^{1}}}_{P_{i,j}}$ can be mapped to the Red channel, ${\bar{KPI^{2}}}_{P_{i,j}}$ can be mapped to the Green channel, and ${\bar{KPI^{3}}}_{P_{i,j}}$ can be mapped to the Blue channel.

The color components are then normalized to fit into an 8-bit integer range, between 0 and 255, to be suitable for the RGB format (or any other format such as YUV or HUE). The transformation for each color component can be represented as follows:

(5) $$R_{P_{i,j}}=255\;\cdot \;\frac{{\bar{KPI^{1}}}_{P_{i,j}}-\min\left(\bar{KPI^{1}}\right)}{\max\left(\bar{KPI^{1}}\right)-\min\left(\bar{KPI^{1}}\right)}$$

(6) $$G_{P_{i,j}}=255\;\cdot \;\frac{{\bar{KPI^{2}}}_{P_{i,j}}-\min\left(\bar{KPI^{2}}\right)}{\max\left(\bar{KPI^{2}}\right)-\min\left(\bar{KPI^{2}}\right)}$$

(7) $$B_{P_{i,j}}=255\;\cdot \;\frac{{\bar{KPI^{3}}}_{P_{i,j}}-\min\left(\bar{KPI^{3}}\right)}{\max\left(\bar{KPI^{3}}\right)-\min\left(\bar{KPI^{3}}\right)}$$

The specific transformation function can be further refined by using feature engineering techniques to identify the most relevant indicators to map onto the color components. Additionally, advanced image processing techniques can be employed to enhance the visual perception of the synthetic image, facilitating further analysis.

The NSI generation process can be summarized in the pseudo-code algorithm provided in Algorithm 1.
**Algorithm 1:** NSI Generation.**Input:** Δt {Temporal resolution (seconds)}, Δp {Spatial resolution or pixel size (meters)}, Geolocated traces from UEs**Output:** Network State synthetic Image**procedure** GenerateNSI     **Pixelize** the area of interest using defined spatial resolution.     **for** each UE measurement $LM_{UE_{i}}\left(T\right)$ in the collected data **do**          **Locate** the UE in the corresponding pixel $P_{i,j}$ based on geographical location.          **Add** the UE’s KPI measurements $KPI_{UE_{i}}\left(T\right)$ to the set of LMs $LM_{P_{i,j}}$ for that pixel.     **for** each pixel $P_{i,j}$ in the pixelized area **do**          **MergeLM**($LM_{P_{i,j}}$) to calculate the aggregated KPI values ${\bar{KPI^{1}}}_{P_{i,j}}$, ${\bar{KPI^{2}}}_{P_{i,j}}$, and ${\bar{KPI^{3}}}_{P_{i,j}}$ using statistical method (e.g., average).          **LMCoding**(${\bar{KPI^{1}}}_{P_{i,j}}$, ${\bar{KPI^{2}}}_{P_{i,j}}$, ${\bar{KPI^{3}}}_{P_{i,j}}$) to calculate the image format values $R_{P_{i,j}}$, $G_{P_{i,j}}$, and $B_{P_{i,j}}$ for the pixel using normalization.     **GenerateNSI** the synthetic image by combining image format values for all pixels.

A key challenge with DL methods lies in their requirement for a vast quantity of training images. The proposed approach addresses this by systematically collecting geotagged and timestamped traces over varied time periods. This allows for a robust collection of NSIs to be created from this comprehensive and diverse dataset. NSIs serve as precise depictions of different cellular network operating conditions, offering a rich training dataset for DL models.

This method enables detailed network state representation, accounting for both spatial and temporal dimensions. Balancing spatial and temporal resolution is vital to avoid data overloading and visual clutter. Adjusting these variables can create a more thorough, relevant, and accurate network coverage image. This image can significantly aid in identifying and resolving network problems, enhancing overall performance and user experience.

A critical aspect of this approach is the labeling of NSIs. Here, expert knowledge is pivotal, with network specialists providing labels based on their deep understanding of network states or failures. This process helps establish a reliable ground truth for training DL models, enhancing their ability to interpret new NSIs in an operational setting accurately.

In some cases, specific failure data may not be readily available, which presents a challenge for labeling. To overcome this, the approach can employ artificial failure traces creation [27]. Using established radio propagation and failure models, simulated failures can be incorporated into NSIs, allowing for a more representative dataset. This innovative method enables DL models to learn and identify a broader range of failure states, improving their predictive capabilities and overall performance.

Moreover, its compatibility with unsupervised learning methods demonstrates the proposed approach’s versatility. While supervised learning with labeled NSIs can provide excellent results, unsupervised methods also have unique advantages. By applying unsupervised techniques to NSIs, the system can extract valuable insights and hidden patterns from data without explicit labeling. This feature further enhances the proposed approach’s flexibility and strength, making it a versatile tool for handling 6G network complexities.

### 3.2. Deep Learning Inference

Once a sufficient collection of NSIs has been established, these can serve as inputs for DL image analysis techniques. The choice of a specific DL algorithm largely hinges on the intended management task and the proper construction of the NSIs. The accuracy and efficiency of the analysis will be influenced by visual aspects such as shape and color.

Among potential DL algorithms, Convolutional Neural Networks (CNNs) have revolutionized image classification, often delivering high accuracies. CNNs take an image as input, process it, and categorize it into specific groups. This process relies on “convolution”, a technique that scans an image with a filter to produce a new, processed image. The network learns image features, starting from minute details in the initial layers to more generalized image features in the final layers. The CNN identifies image components by learning from a variety of filters across multiple layers; convolution pinpoints local patterns, while pooling detects global ones.

CNNs are particularly effective at image classification due to their inherent ability to form spatial hierarchies of features through repeated application of convolutions and pooling operations. To tailor a CNN for specific network management tasks, such as self-diagnosis, the architectural design and the training dataset must be adapted. We explore these adaptations in more detail below:

- **Visual Characteristics:** NSIs can depict specific sectors within the mobile network environment at a pixel level. Different color gradients or intensities correspond to radio parameters such as RSRP, RSRQ, and SINR, among other performance indicators. These visual traits encapsulated within the NSIs reflect various aspects of network performance and serve as input features for CNN.
- **Adaptation of CNN Architecture:** The CNN architecture, aimed at network self-diagnosis, needs to be structured to capture pertinent features within the NSIs. This involves:
  – Convolutional Layer Selection: The number, dimensions, and stride of the convolutional filters should be chosen wisely to extract spatial features from the NSIs.
  – Pooling Layers: Pooling layers can retain key features while reducing the spatial dimensions of the NSIs. In a self-diagnosis task, max pooling can be implemented to preserve the most significant signal features during the dimensionality reduction phase.
  – Fully Connected Layers: Given the dynamic and diverse 6G network environment, the number of neurons and layers within the fully connected part of the network needs to be optimized to handle the nuanced complexity of potential interference or power issues.
  – Output Layer: The output layer should be designed to provide the required classification output, such as identifying areas of weak signal quality or deducing the root cause of network failures.

In NSIs, the colors or intensities can represent varying measured radio values, as well as indicators such as dropped calls or data throughput. Once synthesized, these NSIs can be used to train the CNN to classify regions into categories such as “good coverage”, “poor coverage”, “interference”, or “congestion”. Image classification DL algorithms can identify patterns beyond human perception, potentially highlighting areas of concern. An essential use case involves using historically labeled data to recognize network issues during a past failure or the preceding hours. This predictive capability enables the algorithm to provide proactive alerts to prevent network failures.

The CNN architecture can be tailored to this problem using multiple convolutional layers with filters designed to capture spatial network performance patterns. Pooling layers could be added to help the network focus on larger-scale patterns, while fully connected layers could be tuned to provide the desired level of abstraction. The output layer could be designed to classify each region, enabling the network provider to identify and address performance issues rapidly. A set of specific hyperparameters: such as the number of layers, activation function, epoch, or batch size, in addition to traditional weights and biases: allows for fine-tuning, potentially leading to high classification accuracy.

However, it’s worth noting that while CNN is the preferred DL algorithm for image classification in our framework, the approach is not strictly confined to it.

## 4. Evaluation

The effectiveness and versatility of the proposed framework are assessed through the use of a comprehensive scenario adopted from [27].

This is facilitated by a system-level simulator that has undergone rigorous validation. The NSIs resulting from this scenario are shown in Figure 2. The scenario is inspired by the departure area of Malaga City Airport, which is characterized by its complex architectural design, including walls and boarding gates.

The challenging environment includes 12 picocells scattered over an area of 200 × 300 m, complemented by three macrocells, the closest of which is located approximately 500 m north-west of the highlighted building area. The validation of the results is strengthened by the use of the UMA-developed LTE simulator [28], which has been extensively supported in various studies.

Users in the simulated scenario explore all reachable areas, replicating regular user density patterns at designated locations, such as security checkpoints and boarding gates. Network measurements encapsulate cell-level metrics and direct UE radio reports, reflecting those in actual deployments.

Radio-based indicators allow a quick response to possible network faults, eliminating the need for large numbers of users or long measurement durations. Key Performance Indicators (KPIs) are calculated as the average of collected UE measurements during each 60-s simulation loop, with a UE reporting interval of 100 ms. User movements are mimicked by a random waypoint-based model, which outlines user density hotspots in high-impact areas.

Table 2 presents detailed simulation parameters that resemble real-world operational scenarios and provide strong validation of the proposed methodology.

A range of conditions and primary failures are examined, including normal conditions (no failures), macrocell interference, picocell interference, and cell power degradation. The evaluation of the system involves the analysis of 4004 one-minute network simulation loops, representing almost three days of data. These loops consist of 25% simulating normal network conditions, 25% emulating macrocell interference, and the remainder split between picocell interference and power degradation. The goal is not to provoke network problems, but to create a model that is able to identify and correct naturally occurring problems, thereby boosting network resilience.

A set of NSIs is produced, each employing one UE radio variable per RGB color layer (i.e., RSRP, RSRQ, and SINR), with the intensity being directly mapped based on the normalization of potential values. Three pixel sizes (1, 5, and 10 m) are selected, the choice of which hinges on the accuracy of the localization method in a real environment. As earlier stated, the acquisition time is directly proportional to the pixel size necessary to fill the majority of the image. This condition could lead to an insufficient number of samples for a given pixel size acquisition time, for instance, 10k samples with a 1 m size.

During the evaluation process, different combinations of pixel size (in pixel edge meters) and the total number of NPI samples (ks) are implemented to build NSIs, ensuring that their total area covers the network’s range. This method leads to four configurations: 1 m–100k samples, 5 m–100k samples, 5 m–5k samples, and 10 m–5k samples. The mean value of all samples falling within each pixel is taken to merge data.

A four-layer Convolutional Neural Network (CNN) conducts image recognition, yielding four potential outputs corresponding to the labeled network states. Two baselines are compared: one applying a Naive Bayes Classifier (NBC) to RSRP maps and another utilizing the same map types with a Support Vector Machine (SVM) algorithm, as proposed in [29].

The k-fold cross-validation technique validates the ML models, where ’k’ represents the dataset partitions. Here, ’k’ is set to 5, signifying five-fold cross-validation. Each validation iteration trains the model on k-1 partitions (or folds) and tests it on the remaining one. Every iteration involves training for five epochs, using a batch size of 100.

The models make predictions on the test set after training. These predictions form a confusion matrix, a tabular layout that visualizes an algorithm’s performance through the comparison of actual and predicted classifications. The final performance metric considers the mean performance across all five iterations.

The state-of-the-art SVM method for Radio Frequency (RF) map classification, which exclusively relies on RSRP [29], attains an accuracy below 0.9. In contrast, the NSIs comprising three radio KPIs per LM encoded in RGB colors indicate potential improvement through the proposed framework.

**Figure 3:**
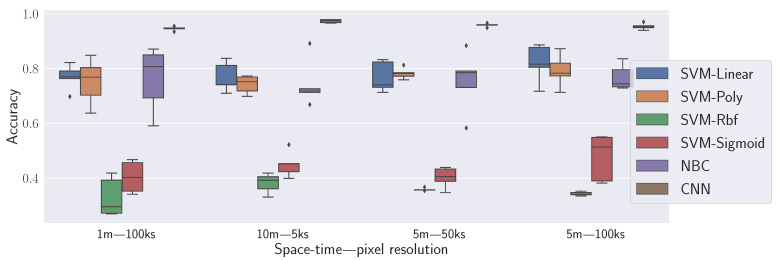
Accuracy comparison.

Evaluation outcomes suggest that changing the hyperparameters C, gamma, and kernel for the SVM-based model per each NSI grouping per scale leads to different results. The most optimal outcome is achieved through the CNN approach at a 10 m scale with only 5000 samples per NSI. This particular configuration exceeds an accuracy of 0.9, a significant accomplishment displayed in Figure 3.

The F1 score formula is utilized to assess the performance of a supervised algorithm. This formula is defined as the harmonic average of precision and recall:

(8) $$F1=2\;\cdot \;\frac{\mathrm{Precision}\;\cdot \;\mathrm{Recall}}{\mathrm{Precision}+\mathrm{Recall}}$$

(9) $$\mathrm{Precision}=\frac{\mathrm{TP}}{\mathrm{TP}+\mathrm{FP}}$$

(10) $$\mathrm{Recall}=\frac{\mathrm{TP}}{\mathrm{TP}+\mathrm{FN}}.$$

Here, TP stands for true positives, FP for false positives, and FN for false negatives.

**Figure 4:**
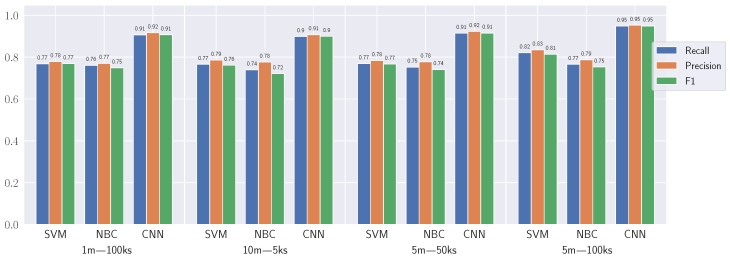
Performance comparison of image classification methods.

As confirmed in Figure 4, the generic CNN with RGB images surpasses alternative methods on all diagnosis indicators (precision, recall, F1), achieving a performance above 90% in all cases. A 5m spatial resolution is determined to provide optimal output for the CNN algorithm, considering the reduced computational cost associated with lower spatial resolution.

The application of NSIs derived from location data allows for the effective usage of image classification algorithms from low-precision positioned traces. Such NSIs can be further refined according to their intended application.

**Figure 5:**
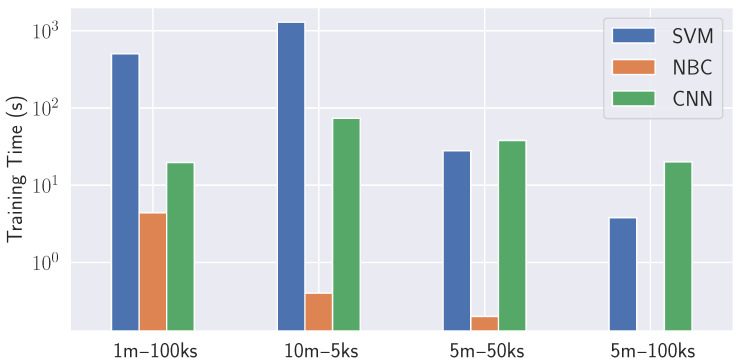
Training time comparison.

In terms of training time, the proposed image-based CNN approach is superior to SVM and NBC. All algorithms and configurations are tested on an Intel Xeon processor server with eight cores and a processing speed of up to 2.4 GHz per core. As illustrated in Figure 5, CNN demonstrates shorter training times compared to SVM and NBC, barring the 10 m–5 ks configuration, where they are almost equivalent.

Although network states are equally represented in this scenario, data imbalance can present a considerable challenge, especially when handling rare fault states. The underrepresentation of these fault states in the dataset could affect the model’s performance. Various strategies could be employed to address this issue. For instance, the creation of artificial data based on available instances of these states can increase their representation and aid the model in understanding these states better.

Additionally, Synthetic Minority Over-sampling Technique (SMOTE) [30] could be utilized to handle data imbalance. It works by selecting examples that are close in the feature space, drawing a line between the examples in the feature space, and drawing a new sample at a point along that line.

Alternation of the loss function can serve as a promising strategy to overcome this challenge. By customizing the loss function to penalize the misclassification of less common states more severely, the model can be influenced to concentrate more on these underrepresented states during the learning process.

Utilizing Transfer Learning, a method that applies insights from pre-trained models can effectively mitigate data imbalance. By leveraging a model previously trained on a more extensive and diverse dataset, transfer learning can boost the model’s performance on the less represented fault states.

Active learning is a further approach that can assist in countering data imbalance. In active learning, the model participates in the data collection procedure, allowing it to request more samples of the underrepresented states if needed. This process can contribute to a more balanced dataset and, consequently, the enhanced performance of the model.

Finally, ensemble methods, which amalgamate the predictions of several models, can aid in improving the predictive performance on infrequent fault states. Ensemble methods can offset the limitations of individual models, resulting in a more sturdy and balanced predictive capability.

In practical deployment, a feedback loop can be established for continuous model updating with fresh data, enabling real-time optimization and adaptation to changing network conditions. This process, coupled with an integrated approach to data collection [31], diagnosis, and action, can effectively facilitate network self-healing. Considering the diversity and complexity of network issues, continuous training with new problem instances is necessary to comprehensively cover all potential faults and their variations, which might require regular model updates using newly collected data, the frequency of which depends on the network size, its rate of change, and available computational resources.

## 5. Conclusions and Outlook

The work presented highlights the significant promise of Network Synthetic Images as an innovative tool for managing complex 6G mobile networks. The foundations of an NSI generation framework, formed from geo-localized traces, have been delineated, shedding light on the associated factors and their consequential impact on the processing timeline. The verification of the effectiveness of the developed approach in network fault management illustrates the precision of network state identification enabled by the careful optimization of image samples and coverage areas. The proposed framework, which integrates CNN-based classifiers for fault diagnosis, is shown to outperform traditional SVM-based approaches using RSRP map samples.

The technique of creating synthetic images from KPI geo-localized traces, coupled with advanced image classifiers, paves an optimistic path towards the evolution of automated 6G network management. This method may potentially refine the currently complex network management processes that require intricate log analyses by specialists, further enabling the discovery of previously undetected issues.

Overall, the results demonstrate the promising capability of the proposed framework in using location-based network data to predict and diagnose potential network issues effectively. The efficient and accurate diagnosis of faults, coupled with a low computational cost, makes this framework an attractive solution for improving the resilience of future networks.

Despite the optimistic results, several opportunities for future exploration have been discerned to improve NSIs’ efficacy and application range. Introducing additional data sources such as user mobility patterns or environmental factors may provide a more holistic representation of the cellular network, potentially increasing the accuracy of NSIs and overall network management.

Continued exploration into advanced deep learning models such as Deep Neural Networks (DNN) or Recursive Neural Networks (RNN) may refine NSI generation and interpretation, potentially yielding superior performance in network diagnosis tasks. The proposition for future exploration also includes alternative management techniques that utilize geolocated mapping. The potential issue of data inadequacy might be alleviated by employing Generative Adversarial Network (GAN) techniques [32]. Future exploration could focus on feature engineering [33] to enhance the representativeness of NSIs, placing emphasis on the distinctive features of an area to visually augment its significance.

Expanding the NSI framework to include multiple KPIs or other network performance metrics in a single synthetic image could provide a broader view of the network’s state, thereby aiding the identification of complex problems or correlations among different network elements. The utilization of image treatment algorithms, such as filters for NSI post-processing, might enhance visual cues, thereby facilitating optimal training of machine learning mechanisms [34].

The development of methodologies for real-time updates of NSIs as fresh data become accessible could pave the way for more dynamic and adaptable network management, fostering swift responses to changes in the network environment. An evaluation of the proposed framework’s scalability for large and heterogeneous networks, particularly the rapidly advancing 6G networks, is imperative to ensure future usability.

Finally, the analysis of NSIs over time (in a multimodal format such as a movie) may provide network operators with invaluable insights for real-time monitoring, predictive analysis, automatic network diagnosis, and optimization. This could unlock a more efficient and zero-touch network management paradigm, fostering a seamless transition into the era of 6G.

## Figures and Tables

**Table 1:** Summary of works on ML for Radio Environment Maps.

Reference	Method Proposed	Source of Information	Application	Gaps Identified
[13]	K-means clustering, DBSCAN	RSS measures	Spectrum management	Dynamic nature of the wireless environment.
[14]	SVM, decision trees	RSS measures	Spectrum sharing	Heterogeneous nature of the wireless environment.
[15]	Gaussian mixture model	RSS measures	Spectrum sensing	Uncertainty of the wireless environment.
[20]	Siamese neural networks, attention mechanism	Image data	REM updating	Scalability of the algorithm.
[19]	Bayesian network	RSS measures	REM construction	Accuracy of the algorithm.
[16]	Genetic algorithm, DenseNet	Image data	Image classification	Robustness of the algorithm to noise.
[21]	Self-labeling	RSS measures	REM construction	Security of the algorithm.
[17]	Spatial statistics, Bayesian hierarchical model	RSS measures, CSI	REM construction	Temporal dynamics of the wireless environment.
[18]	Spatial statistics, Bayesian model	RSS measures, geolocation data	REM construction	Spatial correlation of the wireless environment.
[22]	Spatial statistics, Deep Neural Networks	RSS measures, signal strength data from multiple sources	REM estimation	Accuracy of the algorithm in different environments.
[23]	Artificial intelligence, Internet of Things, 5G	Smart grid	Next-generation smart grid	Cost-effectiveness of the solution.

**Table 2:** Simulation parameters.

Parameter	Detail	Value
LTE Simulator Time resolution	Model	100 TTI (100 ms)
RRM model	Cell reselection	Criteria S, R
	Handover	Events A3, A5
Traffic model	Calls	Poisson (avg. 0.43 calls/user·h)
	Duration	Exponential (avg. 100 s)
Mobility model	Outdoor	3 km/h, random direction & wrap-around
	Indoor	Random Waypoint based model with hotspots
UE model	Noise figure	9 dB
	Noise density	−174 dBm/Hz
Base station	Directivity	Omni (small)/tri-sector (macro)
	Access	Open (small)/open (macro)
	Equivalent Isotropically Radiated Power (EIRP)	3 dBm (small cells)/43 dBm (macro)
Propagation model	Indoor–indoor	Winner II A1
	Indoor–outdoor	Winner II A2
	Outdoor–outdoor	Winner II C2
	Outdoor–indoor	Winner II C4

## Data Availability

Not applicable.

## Abbreviations

The following abbreviations are used in this manuscript:

5G	Fifth Generation Technology
6G	Sixth Generation Technology
CNN	Convolutional Neural Network
DBSCAN	Density-Based Spatial Clustering of Applications with Noise
DL	Deep Learning
DNN	Deep Neural Networks
FN	False Negatives
FP	False Positives
GAN	Generative Adversarial Network
GNSS	Global Navigation Satellite Systems
IoT	Internet of Things
K-means	K-means Clustering
KPIs	Key Performance Indicators
LCS	Localization Services
LMs	Localized Measurements
ML	Machine Learning
NSI	Network Synthetic Images
PoC	Proof-of-Concept
REM	Radio Environment Map
RF	Radio Frequency
RGB	Red Green Blue
RIS	Reconfigurable Intelligent Surfaces
RNN	Recursive Neural Networks
SVM	Support Vector Machine
TP	True Positives
UE	User Equipment
YUV	Luminance and Chrominance
ZT	Zero-Touch
